# Using an intersectionality approach to transform health services for overlooked healthcare users and workers after covid-19

**DOI:** 10.1136/bmj-2022-072243

**Published:** 2023-06-07

**Authors:** Mamothena Mothupi, Jashodhara Dasgupta, Seyede Sedighe Hosseini Jebeli, Jacqui Stevenson, Karla Berdichevsky, Sreytouch Vong, Edwine Barasa, Asha George

**Affiliations:** 1 School of Public Health, University of the Western Cape, Cape Town, South Africa; 2 Nainital, India; 3 Population Health Science Institute, Newcastle University, Newcastle upon Tyne, UK; 4 United Nations University International Institute of Global Health, Kuala Lumpur, Malaysia; 5 National Center for Gender Equity and Reproductive Health, Ministry of Health, Mexico City, Mexico; 6 Phnom Penh, Cambodia; 7 Health Economics Research Unit, KEMRI-Wellcome Trust Research Programme, Nairobi, Kenya; 8 Complexity and Social Change, School of Public Health, University of the Western Cape, Cape Town, South Africa

## Abstract

Intersectional analysis and action are needed to prepare for future pandemics and ensure more inclusive health services, say **Mamothena Mothupi and colleagues**

Globally, government responses to the covid-19 pandemic reinforced prevailing patterns of privilege and prejudice and further entrenched the inequitable distribution of health and disease in different populations.^1^ ^2^ ^3^ These patterns reflect how the legacies of historical discrimination combine with existing power structures to shape, condone, and continue social disadvantage and the unequal distribution of resources. Analysis of these inequalities within health systems from the perspective of intersectionality can help us understand their drivers and find solutions to reduce them. Tackling these inequalities can also help transform health services for improved pandemic preparedness.^4^ ^5^ ^6^

Intersectionality “promotes an understanding of human beings as shaped by the interaction of different social locations, for example, race, ethnicity, indigeneity, gender, class, sexuality, geography, age, disability/ability, migration status, religion. These interactions occur within a context of connected systems and structures of power, for example, law, policies, state governments, religious institutions, and the media. Through such processes, interdependent forms of privilege and oppression shaped by colonialism, imperialism, racism, homophobia, ableism, and patriarchy are created.”^7^ Whether intentionally or unintentionally, these processes lead to combined and hence deeper inequalities (for example, unmarried minority adolescent girls who are also refugees), which vary by time and context and have consequences for the effectiveness of health services, particularly during crises.

The World Health Organization recommends ensuring healthcare access for marginalised populations and support for the health workforce as a key part of pandemic preparedness and responsiveness.^8^ In this paper, we use intersectionality to better understand the health inequalities that characterised covid-19 and put forward principles for making post-covid-19 health services more responsive to correcting such inequalities. We argue that health services must be more intentionally inclusive, guard against unintended exclusionary consequences of health measures, and invest in research and data systems to better understand and respond to intersectional inequalities. Key to these efforts is more meaningful action to empower the people at the core of the cocreation of health: healthcare users and workers. Their participation is particularly important if we are to tackle the intersectional inequalities exposed by the pandemic, which continue to undermine health and justice.

## Health service delivery must be intentionally inclusive

Delivery of health services during the covid-19 pandemic was generally characterised by top-down approaches to decision making. Government evaluations of policy responses in 18 countries of the Organisation for Economic Co-operation and Development during the first 15 months of the pandemic commended various timely and positive responses. However, the evaluations also revealed challenges in health service delivery and access, including inadequate coordination with local actors on testing and contact tracing for covid-19, poor communication with the public on health service continuity, and limited government capacity to adequately identify and provide resources for vulnerable people.^9^ These policy responses were not only disconnected from the realities on the ground, but were also exclusionary to people with multiple context specific disadvantages, many of whom already distrusted the government because of their history of exclusion and discrimination. Recognising and resolving such disconnects is not new.

Understanding the perspectives of marginalised communities, their contextual priorities and customs, and fears given their prior interactions with the state was essential in past outbreaks^10^ and with covid-19 as well.^11^ ^12^ As such, WHO recommends community engagement as crucial to fight disinformation during and after pandemics.^8^ Beyond times of crisis, participatory models for interventions are effective in improving health,^13^ and WHO has detailed guidance on how community views, participation, and empowerment support expanding universal health coverage.^14^

Marginalised community members who needed health services were not the only ones excluded from pandemic decision making and planning. Lower tier health workers, who often experience multiple, intersecting forms of marginalisation, were also excluded from these processes. For example, community health workers (CHWs) in Brazil, who are predominantly black, female, and working class, were effectively left out of early pandemic responses that prioritised clinical services. As a result, these health workers were left with less access to personal protective equipment (PPE), training, testing services, and supervisory support.^15^ ^16^ The resulting risky working conditions made it impossible for the CHWs to interact with communities and ensure continuity of services. This situation therefore jeopardised a key community resource valued by marginalised users who rely on CHWs because of the many barriers to access of health services that they face, which were made worse by covid-19.^17^ Insufficient attention to the needs of CHWs in the covid-19 response was common in many countries.^18^

Given the increased risks and stresses health workers were exposed to in different countries, they led advocacy efforts to improve their working conditions. In a few instances, their efforts resulted in important changes. In Spain, hospital cleaners were able to organise and assert their right for vaccination as part of the at-risk category of people prioritised for vaccination.^19^ In South Africa, CHWs were last on the list for receiving PPE, but through their self organising forums and unions, they began strikes and protests to fight to change this situation.^20^ In Malawi, as a result of sit-ins in central and district hospitals and civil society activism, more than 12 000 CHWs in hard-to-reach areas of the country were finally provided with PPE.^21^ In India, hundreds of thousands of accredited social health activists (ASHAs) (female CHWs) organised sit-ins, protesting their delayed payments, lack of health coverage, and undervalued contributions to the pandemic response.^22^

Throughout efforts to give voice to the most marginalised people and ensure they are included in pandemic responses, researchers and public health decision makers also need to consider: which groups are engaged; what the power hierarchies are that configure these groups; whether their actions are creating or entrenching disadvantage and lack of trust; and whose voices are not represented.

## Health services may exclude or discriminate in unintended ways

In responding to covid-19, many governments did in fact implement policies and strategies to try to tackle inequalities in access to and delivery of health services. However, the benefits were not always available to everyone. For example, in southern Rajasthan, India, health and social protection policies related to the pandemic were implemented to mitigate the effects of covid-19 and covid-19 lockdown measures.^23^ However, if the policies considered women’s needs, they did so on the assumption that the experiences of all women were the same. But, some migrant women and female headed households were still inadequately reached because they were particularly disadvantaged due to illiteracy, economic constraints, restrictions on mobility, poor representation in local government, and corrupt service providers, in addition to patriarchal norms.^23^ Thus, even when inclusive policies exist, an intersectional approach requires time and flexibility for service managers to actively monitor its effect on marginalised groups and, in consultation with end users, proactively develop innovative changes to overcome the barriers created by enduring unfair systems.

Similarly, human resources reforms within the health sector, including those produced by advocacy efforts of health workers, do not always result in benefits for all health workers.^19^ ^20^ ^21^ While the terms of employment and challenging working conditions of nurses have been widely discussed, research is lacking on the experiences within this group that are shaped by the intersection of race, ethnicity, gender, and migration among other factors and the measures needed to ensure equity among nurses.^24^ ^25^

While advocacy for improved working conditions for health workers during the covid-19 pandemic had some success, overall unionisation is less common as one moves down the health workforce hierarchy, whether in terms of occupational hierarchy or migrant status.^19^ Furthermore, workers at the lower levels of the health workforce are more likely to face government backlash when they try to organise efforts to draw attention to their concerns. For example, in India, protesting ASHAs had police reports filed against them.^22^ Beyond applause and symbolic recognition of their contributions, health worker and civil service reforms are required to formalise the work of community and auxiliary workers and increase investment in the health workforce to guarantee equitable baseline work conditions.^16^ ^21^ ^22^ To bring about such reform requires engagement with government across ministries of health and education and medical boards that regulate health workers; investment examples to inform ministries of finance; and advocacy that reframes funding for health worker salaries and working conditions not as a cost but as an investment in an essential asset for pandemic responses and health services in resource constrained settings.

## Research and data systems must prioritise intersectionality 

Research with an intersectionality approach is still rare, however it has the potential to provide an understanding of the negative consequences of pandemic responses on marginalised people and ways to mitigate these consequences. Such research can better indicate who is left out from pandemic responses and why. For example, research in India showed how multiple forms of discrimination impeded access to welfare measures during covid-19 for poor, indigenous (tribal) women.^23^ In Canada, an intersectional analysis of factors that affected access of minority immigrants to emergency/urgent care sought to understand migrant and refugee women’s experience of gender based violence during the pandemic.^26^ ^27^ A cohort study examining the intersectionality of obesity, chronic disease, social factors, and incident risk of covid-19 among low income, middle aged minority mothers in the United States showed who was being left out by current measures and was most at risk of covid-19.^28^ Despite these examples, little primary research has been done that gives contextualised information about how pandemic measures affected health users and health workers. This information could guide intersectional strategies to ensure equitable health services and outcomes for marginalised people.

Health researchers and decision makers are currently limited by the data available from health information systems and surveys which do not lend themselves to intersectionality analysis. Basic sex disaggregated data reported by national health information systems are often lacking. By November 2022, only 85 of 206 countries reported sex disaggregated analysis of confirmed covid-19 cases and 39 reported sex disaggregated data on deaths in the previous two months.^29^ While health information systems do routinely collect data on age and sex, even if they inconsistently report on them,^30^ other sources of data are needed to track broader structural and systemic drivers of inequality.

Policy makers and decision makers responsible for health systems must increase efforts to collect and report disaggregated patient data, as well as strengthen more holistic data collection tools and reporting for human resources for health.^30^ An important first step will be for health system designers and implementers to build capacities within health systems to collect, analyse, and use intersectional data and, importantly, to understand and manage additional ethical concerns about data on vulnerable populations. Newly developed tools to apply intersectionality can also provide a guide to tackling inherent biases within artificial intelligence models and algorithms by incorporating gender and intersectionality in data system investment and data collection, processing, analysis, and use.^31^

Qualitative and quantitative methods can also be used together for a holistic analysis of the intersecting factors that influence health service access and outcomes.^30^ With better data, researchers can analyse intersections of advantage and disadvantage in health access and make recommendations to deal with structural and power dynamics in specific contexts. It is important to go beyond immediate descriptive elements of social factors (for example, age, sex, race, and income) to examine the policies and political and economic drivers of such disadvantages.^32^

## Intersectionality can show the way forward

Current health inequalities arise from historical marginalisation of different groups of people across multiple axes of discrimination. If we continue to ignore these multiple, interconnected, and context specific forms of disadvantage, health services will always fall short on their goals. An intersectionality based approach can help improve pandemic preparedness and response and create better health systems to identify and respond to marginalised health workers and healthcare users.

Healthcare users, and the workers who serve them, are the heart of health systems. To better include marginalised health workers and healthcare users, researchers and decision makers need to consider their complex social positions, the barriers to their engagement with health services, the power systems and structures that create these barriers, and how to overcome them. This approach would empower healthcare users and health workers and allow them to advocate for a transformed and inclusive system that leaves no one behind. As a way forward, governments and health system planners should examine and improve their capacities to include marginalised people in future pandemic preparedness and response as part of health systems transformation and justice. Fundamental changes are needed including meaningful engagement with marginalised healthcare users and workers, improved working conditions for marginalised health workers, and improved health intelligence and information systems. These changes, supported by decentralised management to allow innovation locally, would help strengthen health systems for all.

**Key messages**

The effect of intersecting systems of privilege and prejudice on the experiences of healthcare users and health workers during covid-19 must be considered for future pandemic preparedness
Marginalisation is not uniformly experienced and it changes over time, thus health services need to be more inclusive
Using an intersectionality approach is important to improve health system governance capacity to engage with marginalised healthcare users and workers to tackle context specific problems
The views of healthcare users and workers must be meaningfully integrated in the health system transformations needed to prepare for the next pandemic

## Notes

Provenance and peer review: Commissioned; externally peer reviewed.

This article is part of a collection proposed by the United Nations University and the University of the Western Cape and commissioned by *The BMJ*. *The BMJ* peer reviewed, edited, and made the decision to publish these articles. Article handling fees are funded by Bill and Melinda Gates Foundation and UN University-International Institute for Global Health.
